# A Conceptual Framework for Preparing Residents to Broach Racial, Ethnic, and Cultural Factors with Patients in Supportive Psychotherapy

**DOI:** 10.1007/s40596-023-01802-9

**Published:** 2023-05-31

**Authors:** Mary Beth Cogan, Clio Franklin, Norma L. Day-Vines, Donna M. Sudak, Zane Sejdiu, Yash Patel, Hal Kronsberg, Anne Ruble

**Affiliations:** 1 Johns Hopkins University School of Medicine, Baltimore, MD USA; 2 Johns Hopkins University School of Education, Baltimore, MD USA; 3 Drexel University, Philadelphia, PA USA

Stigma and discrimination in health care are commonly experienced by ethnic minority populations [1]. The 2021 National Healthcare Quality and Disparities Report indicated that White patients receive better quality of care than 43% of Blacks, 40% of Native Americans, 36% of Hispanics, and 28% of Asians and Pacific Islanders based on indicators of patient safety, person-centered care, care coordination, effective treatment, healthy living, and care affordability [2]. Ignoring, minimizing, or denying racial, ethnic, and cultural issues further marginalize minority populations and perpetuate discrimination [3].

Health care professionals’ cultural responsiveness impacts patient engagement and receptiveness to care [4, 5]. Stigmatizing beliefs lead to a decline in services, resources, and accessibility to care and consequently increased morbidity [6]. Failing to acknowledge and address racial, ethnic, and cultural issues results in barriers to health care [7]. Health care professionals must be trained to increase self- awareness and develop the skills to broach multicultural issues [8].

Educating trainees to recognize and address the social determinants of mental health is critical [9]. The Accreditation Council for Graduate Medical Education specifies sensitivity to diverse patient populations as a core professional competency [10]. Models of conceptualizing culture in psychotherapy include Kleinman’s 8 questions [11], Hays’ ADDRESSING framework [12], and, most recently, the Cultural Formulation Interview (as part of the DSM-5-TR) [13]. The broaching curriculum complements these by focusing on both understanding a model and e-skills training to enhance the therapeutic alliance. A recent survey of child and adolescent psychiatry programs found that no programs teach trainees to incorporate cultural factors in their clinical work in an “extremely effective” way [9]. We must teach clinicians skills to directly address racial, ethnic, and cultural factors with patients [14, 15]. In this educational case report, we describe the implementation of a 6-h curriculum that prepares early psychiatry residents to explore the contextual dimensions of race, ethnicity, and culture with patients in psychotherapy. Our hypothesis was that this curriculum would increase the confidence of residents in navigating issues of racial and other differences in psychotherapy.

## Curriculum Development

A clinician’s appreciation of racial and cultural diversity is not synonymous with the understanding or skill to address these issues in clinical practice [16]. Day-Vines et al. [13, 14] developed a theoretically and empirically supported continuum that identifies stances providers may assume when determining whether to discuss the relevance of race, ethnicity, and culture to patients’ presenting concerns. Broaching is the “deliberate and intentional effort to discuss racial, ethnic, and cultural concerns that may impact a patient’s presenting concerns” and the translation or acknowledgment of the “sociocultural and sociopolitical realities” that impact well-being [15]. The Day-Vines et al. [14, 15] multidimensional model of broaching behavior identifies specific contexts that clinicians may broach and a specific set of strategies that guide the broaching process. Each of these models was adapted to the clinical context of psychiatric residents (e.g., acutely decompensated patients where such conversations may not be feasible). The curriculum was designed to support residents as they acquire skills for discussing patients’ concerns related to their multiculturalism. An introductory lecture provides context regarding the history of American psychiatry, including eugenics and the racial biases that have contributed to the misdiagnosis of African American patients and influence patients’ relationships with health care professionals.

Residents are introduced to the Multidimensional Model of Broaching Behavior, which identifies specific domains of race, ethnicity, and culture that can be explored with patients [1]. The model is illustrated with a case discussion of a biracial patient whose African American father died during his adolescence, who subsequently relocates with his mother to a White working-class rural community. This patient’s educational and career trajectory and subsequent emotional disconnection from his family become the lens through which each dimension of the model is discussed. Following each specific domain, the facilitator provides a video demonstration to enhance understanding.

The “intracounseling dimension” is the first component of the multidimensional model addressing the interpersonal dynamics in the patient-provider relationship related to race, ethnicity, and gender. Residents are taught this component with an example of a broaching intervention [16]:

I know that this can sometimes be a difficult topic to discuss, but I was wondering how you feel about working with someone who is from a different racial/ethnic background? I ask because although it is certainly my goal to be as helpful to you as I possibly can, I also know that there may be times when I cannot fully appreciate your experiences. I want you to know that I am always open to talking about these topics whenever they are relevant [16].

Such a statement communicates that any such concerns are appropriate to discuss and demonstrate the clinician’s cultural responsiveness. Research has demonstrated that exploration of racial, ethnic, and cultural concerns within the clinical encounter leads to heightened levels of self-disclosure [17, 18]. Inattention to the patient’s concerns contributes to cultural concealment and less favorable outcomes [19].

The second component addresses “intraindividual dimensions,” recognizing the complexity of intersectional identities, such as race, gender, sexuality, social class, and accompanying intersectional sources of oppression. In this dimension, the clinician fosters an understanding of how multiple identity dimensions and their interactions influence a person’s experiences, worldview, and health care concerns [13, 14]. The seminar begins with a video demonstrating this skill, followed by broaching exercises related to the case illustration. Residents formulate a broaching statement to inquire about the discomfort the patient experiences around issues of social class status: “It sounds like your experience as an upwardly mobile, cisgendered, biracial man is impacting your family relationships in ways that are different than in the past.” Residents paraphrase the response to validate and convey understanding. The third dimension, “intra-racial-ethnic-cultural concerns,” addresses issues that arise within the groups that the patient is a member. Patients may have beliefs, values, and behaviors that differ from their larger cultural group. Residents are provided an example of a broaching statement: “Could you talk in more depth about your experience as a biracial man who has experienced rejection from family members both during childhood and even now as you achieve career success?”.

The fourth dimension of the model presented, “inter-racial-ethnic-cultural,” explores how sociopolitical forces such as racism, oppression, and discrimination affect individuals. Residents are provided a video example of broaching this topic. An example of an inter-racial-ethnic-cultural statement is “What has it been like for you as a biracial male to work in an organization with limited racial diversity?”.

## Implementation

The study titled “Broaching Issues of Racial and Ethnic Identity in the Clinical Encounter” was reviewed and granted an exemption by the Johns Hopkins Medicine institutional review board. Multidisciplinary faculty presented the virtual 6-h curriculum to 21 2nd-year residents at two general psychiatry training programs in academic medical centers as part of the psychotherapy curriculum. Participation was voluntary. Sessions included didactic lectures, group discussions about the broaching model, and the pre-recorded mock patient supportive psychotherapy encounters between a graduate student and a doctoral-prepared counselor-educator, produced solely for training purposes.

Throughout the seminar series, residents were given a four-stage framework for executing a broaching statement that included strategies, preparation, setting intentions, and formulating a statement while considering the context or specific form of oppression. Residents were encouraged throughout the seminar series to consider dominant sociopolitical forces that result in oppression and discrimination.

## Evaluation

To assess the impact of this curriculum on residents’ confidence and comfort addressing racial, ethnic, and cultural issues with patients, unmatched tests before and after the curriculum were analyzed using the Broaching Attitudes and Behavior Survey (BABS) [20]. The BABS is a self-report measure that operationalizes the Continuum of Broaching Behavior. The instrument contains four subscales that assess openness to broaching. The Avoidant subscale reflects the refusal to broach. The Continuing/Incongruent subscale appraises difficulty broaching. The Integrated/Congruent subscale captures the ability to explore racial, ethnic, and cultural concerns with clients and the Infusing subscale assesses the commitment to social justice and advocacy that eliminate barriers for clients. The instrument uses a 5-point Likert scale: strongly disagree, disagree, neither agree nor disagree, agree, and strongly agree. Subscales are summed and averaged. The subscale with the highest score represents greater endorsement of a particular broaching category. The BABS was administered pre- and post-intervention. Surveys were collected between February and May 2022 via Qualtrics.

The process evaluation of the program included qualitative and quantitative responses to a survey completed via a 4-point Likert response with the options of strongly disagree, disagree, agree, and strongly agree. The survey inquired if the series enhanced residents’ ability to recognize how diversity affects patient care; demonstrate self-reflection; acknowledge differing beliefs and convey respect for diversity; explore diversity in evaluation, treatment, and its influence on interactions; and broach the subjects of race, ethnicity, and cultural differences with patients. Independent samples *t*-test and descriptive data were used for quantitative analysis. Levene’s test was used to determine equality of variance.

Qualitative survey items asked residents to evaluate the quality of the training, examine their attitudes about broaching, and provide suggestions for improvement. Open responses were coded using descriptive and in vivo coding to summarize the response and capture participants’ voices. Responses were clustered when similar content was expressed to capture themes.

## Findings

Twenty-one residents participated in the training. Sixteen residents participated in the pre-test and fifteen residents completed post-test. No data was excluded in the analysis. Residents’ pre-test and post-test scores demonstrated a statistically significant decrease on the continuing/incongruent subscale. The integrated/congruent broaching behaviors revealed a highly significant increase between pre-test and post-test. Residents’ pre-test and post-test avoidant and infusing scores were not significant. Table 1 presents these BABS subscale findings. Table 2 summarizes specific BABS items.

**Table 1:** Residents broaching attitudes and behaviors survey subscale findings pre-post test

Subscale	Pre-test mean (SD) (*n* = 16)	Post-test mean (SD) (*n* = 15)	T-test (two-tailed)
Avoidant	1.9375 (0.15052)	1.6333 (0.11919)	*t*(29) = 1.57, *p* = 0.127
Continuing/incongruent	3.4375 (0.22525)	2.7733 (0.23492)	*t*(29) = 2.041, *p* = 0.050*
Integrated/congruent	2.9896 (0.15401)	3.6600 (0.12362)	*t*(29) = − 3.367, *p* = 0.002**
Infusing	4.2000 (0.15384)	4.4133 (0.13728)	*t*(29) = − 1.030, *p* = 0.312

^*^p < .05, result is statistically significant

^**^p < .01, result is highly statistically significant

**Table 2:** Broaching attitudes and behaviors survey scale select items and distribution pre-test/post-test, mean, and standard distribution

Item	Strongly agree		Agree		Neither agree nor disagree		Disagree		Strongly disagree		Mean (SD)		*t*-test (2 tailed)
	Pre-test	Post-test	Pre-test	Post-test	Pre-test	Post-test	Pre-test	Post-test	Pre-test	Post-test	Pre-test	Post-test	
I experience a sense of awkwardness when I address racial and cultural factors during the counseling process	0	0	6 (37.5%)	5 (33.33%)	2 (12.5%)	10 (66.66%)	5 (31.25%)	0	3 (18.75%)	0	2.69 (1.195)	2.73 (1.000)	0.912
I need a broader range of counseling strategies in order to broach racial and cultural factors more effectively with clients of color	5 (31.25%)	0	7 (43.75%)	8 (53.33%)	1 (6.25%)	7 (46.66%)	3 (18.75%)	0	0	0	3.88 (1.088)	3.00 (1.195)	0.041*
I feel uncertain about my ability to broach cultural factors with clients of color	2 (12.5%)	0	7 (43.75%)	3 (20%)	5 (31.25%)	12 (80%)	2 (12.5%)	0	0	0	3.56 (0.892)	2.53 (0.990)	0.005**
Sometimes I have difficulty identifying a facilitative response once my clients begin to talk about racial and cultural issues	1 (6.25%)	1 (6.66%)	10 (62.5%)	3 (20%)	2 (12.5%)	11 (73.33%)	1 (6.25%)	0	2 (12.5%)	0	3.44 (1.153)	2.73 (1.280)	0.118
Sometimes I have difficulty translating my broaching efforts into a culturally responsive counseling intervention	3 (18.75%)	0	7 (43.75%)	6 (40%)	4 (25%)	9 (60%)	1 (6.25%)	0	1 (6.25%)	0	3.63 (1.088)	2.87 (1.125)	0.066
I generally broach racial and cultural factors throughout my counseling sessions	0	2 (13.33%)	2 (12.5%)	5 (33.33%)	9 (56.25%)	8 (53.33%)	3 (18.75%)	0	2 (12.5%)	0	3.31 (0.873)	2.73 (0.703)	0.052*

^*^p < .05, result is significant

^**^p < .01, result is highly significant

In the program evaluation, residents consistently reported that the seminar enhanced their abilities to recognize how patient diversity affects patient care; demonstrate self-reflection, openness to differing beliefs, and respect for diversity; attend to diversity in evaluation and treatment; and broach the subjects of race, ethnicity, and cultural difference with patients. Residents were in less agreement regarding how the seminar enhanced their ability to identify their beliefs and how those beliefs influenced interactions with patients. Approximately 83% of residents agreed and strongly agreed that the seminar enhanced identification of their beliefs and recognition of the impact those beliefs had on interactions, but 16% disagreed and strongly disagreed with those items.

All residents agreed that the broaching curriculum should be integrated into future psychotherapy training. An example of feedback indicated, “Race is difficult to discuss, and this shows why it is important to address. [It is] great for individuals with more privilege to foster understanding of racial injustice/bias. The language is really helpful. [The curriculum] offers tools and resources.”

The most prevalent themes that residents described as helpful were the “practical language” and critical skills broaching afforded them. They expressed diminished fears of “getting it wrong” after being “given permission” to bring up diversity issues and felt reassured that their authenticity was a counterweight. They described the video observation and hearing their peers’ experiences as helpful. They valued broaching as intentional, direct, and conceptualized across four dimensions. They related understanding that diverse patients experience unique challenges and that they represent not only themselves but a larger group and system.

Contrasting opinions were expressed about the frequency of role-play. There was an opinion that the content had not felt “geared to me,” referencing terms such as *client* versus *patient* and that videos presented a patient who was “articulate, culturally aware, not seriously mentally ill,” and in a long-term therapeutic relationship and, therefore, different from their clinical experience. The most prevalent theme to emerge was the need for consolidation of content specific to psychiatry within each dimension of the model connecting broaching with clinical presentation, diagnosis, care, and phrasing tips.

## Discussion

This seminar taught resident skills to broach diversity differences within the context of supportive psychotherapy. Following the training, there was a highly statistically significant decrease in residents’ uncertainty about broaching cultural factors, and a statistically significant increase in residents broaching racial and cultural factors throughout counseling. There was a statistically significant decrease in residents reporting that they needed a broader range of counseling strategies to broach issues of race and culture. Our survey responses were not powered to draw conclusive findings and the findings and the focus of analysis were descriptive. Our subscale findings may suggest that residents’ responses were becoming more nuanced, and that they were becoming more self-aware of their skill level. There was not a significant increase in residents’ infusing broaching behavior or advocacy within the system which may be consistent with their junior status.

While these descriptive findings are encouraging, they need to be interpreted with caution given the small sample size, potential bias among participants, and need for replication. Furthermore, this educational case report is the first use of the BABS as an evaluation measure. In the past, it has been used to explore the dimensionality of the model, so no pre- and post-intervention comparisons can be drawn with other professions. Despite these limitations, future educational interventions may build upon this initial effort to present a model of broaching that can be used to improve residents’ comfort and skills with directly addressing racial, ethnic, and cultural issues with patients.

Recognizing the benefits of this seminar, we are committed to its ongoing integration within our training programs. It is critically important that physicians develop skills to engage in meaningful conversations with diverse patients about their lived experience and its impact on their health care. Educational efforts that foster cultural sensitivity, humility, and respect can be used to strengthen skills and foster more robust therapeutic alliances.
